# Supplementary material for: Adenosine signalling to astrocytes coordinates brain metabolism and function
Source: Nature. 2024 Jul 3;632(8023):139–46. doi: 10.1038/s41586-024-07611-w (PMC11291286; doi:10.1038/s41586-024-07611-w)
Supplement: Supplementary file 1 — Gel source data underlying Fig. 2c. Beta-actin immunoreactivity was detected on the same gels and used as loading control [file 41586_2024_7611_MOESM1_ESM.pdf]

nature portfolio

https://doi.org/10.1038/s41586-024-07611-w

Supplementary information

Adenosine signalling to astrocytes coordinates brain metabolism and function

In the format provided by the authors and unedited

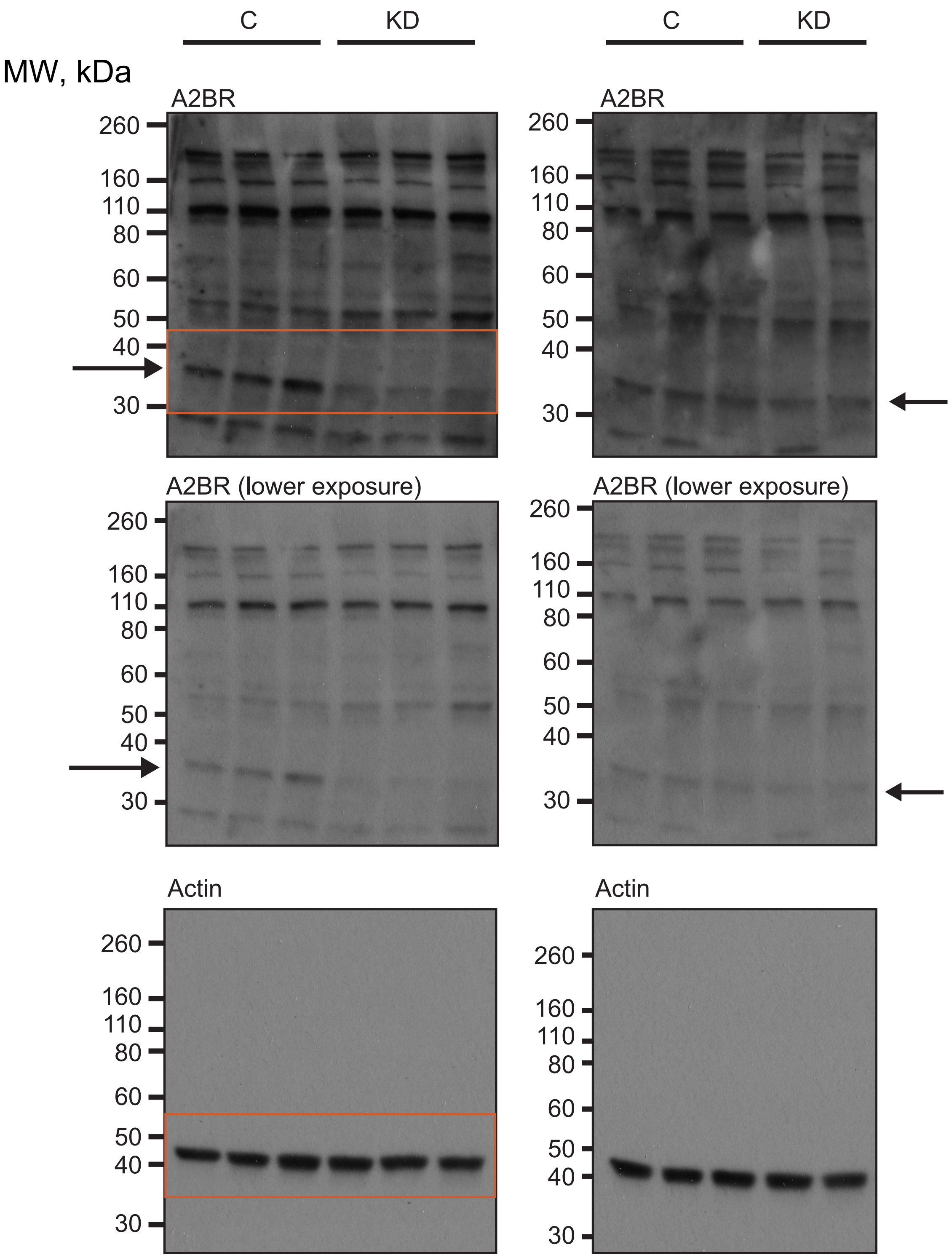

Supplementary Figure 1: Gel source data underlying Fig. 2c. Beta-actin immunoreactivity was detected on the same gels and used as loading control.
