# Supplementary material for: Adenosine signalling to astrocytes coordinates brain metabolism and function
Source: Nature. 2024 Jul 3;632(8023):139–46. doi: 10.1038/s41586-024-07611-w (PMC11291286; doi:10.1038/s41586-024-07611-w)
Supplement: Supplementary file 2 — Reporting Summary [file 41586_2024_7611_MOESM2_ESM.pdf]

nature portfolio

Corresponding author(s): Alexander Gourine

Last updated by author(s): Apr 29, 2024

Reporting Summary

Nature Portfolio wishes to improve the reproducibility of the work that we publish. This form provides structure for consistency and transparency in reporting. For further information on Nature Portfolio policies, see our Editorial Policies and the Editorial Policy Checklist.

Statistics

For all statistical analyses, confirm that the following items are present in the figure legend, table legend, main text, or Methods section.

n/a	Confirmed	
☐	☒	The exact sample size (*n*) for each experimental group/condition, given as a discrete number and unit of measurement
☐	☒	A statement on whether measurements were taken from distinct samples or whether the same sample was measured repeatedly
☐	☒	The statistical test(s) used AND whether they are one- or two-sided<br>*Only common tests should be described solely by name; describe more complex techniques in the Methods section.*
☒	☐	A description of all covariates tested
☐	☒	A description of any assumptions or corrections, such as tests of normality and adjustment for multiple comparisons
☐	☒	A full description of the statistical parameters including central tendency (e.g. means) or other basic estimates (e.g. regression coefficient) AND variation (e.g. standard deviation) or associated estimates of uncertainty (e.g. confidence intervals)
☐	☒	For null hypothesis testing, the test statistic (e.g. *F*, *t*, *r*) with confidence intervals, effect sizes, degrees of freedom and *P* value noted<br>*Give P values as exact values whenever suitable.*
☒	☐	For Bayesian analysis, information on the choice of priors and Markov chain Monte Carlo settings
☒	☐	For hierarchical and complex designs, identification of the appropriate level for tests and full reporting of outcomes
☒	☐	Estimates of effect sizes (e.g. Cohen's *d*, Pearson's *r*), indicating how they were calculated

Our web collection on statistics for biologists contains articles on many of the points above.

Software and code

Policy information about availability of computer code

Data collection	Axon-pClamp (version 10.2); Spike2 (Cambridge Electronic Design Ltd, version 7); IQ3 imaging (version 6.3); Micromanager (ImageJ, version 1.4.23); Olympus FluoView (version 4); Sirenia Acquisition (Pinnacle, version 2.2.7); Skyline (MacCoss Lab Software, version 23.1); VisionWorksLS (Bio-Rad).
Data analysis	Axon-pClamp (version 10.2); GraphPad Prism (version 8); Image J (version 1.52P); MetaboAnalyst R package (version 5.0); MetaboAnalyst R package (version 5.0); Origin 2019 (version 9.6); ropls R package (version 1.26.4); Seurat package (version 4.2.2, "Innocent and Trusting") in R (version 3.6.0, "Planting of a Tree"); Skyline (version 23.1); SleepSign (Kissei Comtec, version 3.0); Spike2 (version 7); Viewer III software (Biobserve, version 3).

For manuscripts utilizing custom algorithms or software that are central to the research but not yet described in published literature, software must be made available to editors and reviewers. We strongly encourage code deposition in a community repository (e.g. GitHub). See the Nature Portfolio guidelines for submitting code & software for further information.

Data

Policy information about availability of data

All manuscripts must include a data availability statement. This statement should provide the following information, where applicable:

- Accession codes, unique identifiers, or web links for publicly available datasets
- A description of any restrictions on data availability
- For clinical datasets or third party data, please ensure that the statement adheres to our policy

The data that support the findings in this study are included within the Supplementary Material. The source data underlying Figs. 1b, e, f, 2c, f, g, i, 3c, d, f, g, h, i, l, 4b, c, e, f, g, h, 5c, d, f, i, and Extended Data Figs. 1e, 2d, e, 3c, 4a, b, 5b, c, d, e, f, 6, 7a, b, 8a, b, c, and 9c, d are provided as Source Data files. Single cell RNAseq source data underlying Fig.2a,b are available from a publicly available database (http://mousebrain.org/). The isotopically quantified LC–MS/MS data are deposited in MassIVE and available via the link https://doi.org/10.25345/C5X05XQ2B (accession number MSV000094445).

Human research participants

Policy information about studies involving human research participants and Sex and Gender in Research.

Reporting on sex and gender	Not applicable.
Population characteristics	Not applicable.
Recruitment	Not applicable.
Ethics oversight	Not applicable.

Note that full information on the approval of the study protocol must also be provided in the manuscript.

Field-specific reporting

Please select the one below that is the best fit for your research. If you are not sure, read the appropriate sections before making your selection.

☒ Life sciences ☐ Behavioural & social sciences ☐ Ecological, evolutionary & environmental sciences

For a reference copy of the document with all sections, see nature.com/documents/nr-reporting-summary-flat.pdf

Life sciences study design

All studies must disclose on these points even when the disclosure is negative.

Sample size	The paper describes the results of the experiments performed using in vitro (primary culture, organotypic and acute brain slices) and in vivo animal preparations. Power calculations for the in vivo animal studies are described below. The significance was set at 0.05 and the beta was set at 0.20. From years of relevant research experience, we expect to detect a significant difference with a minimum of 5 animals per experimental group, if the treatments cause differences between means that are as large as 2.25 standard deviations (SD), likely to be a physiologically significant difference. If the difference is as small as 1.75 SD, we increase sample sizes to 9. In the in vitro studies, the data in individual experiments were collected from a minimum of 3 different slices or cultures (termed 'samples' in the Source Data files) prepared from the same number of different animals. In each experimental preparation (sample), the recordings were made from several individual cells. This experimental design is based on many years of research experience in conducting experiments of this type. Statistical analysis was conducted on the collected data, taking into account the number of cells in each experimental sample and the number of biologically distinct samples. No specific statistical tools were used to predetermine the sample size. The sample sizes were chosen to provide sufficient statistical power to detect biologically significant differences between experimental treatments, considering the experimental design and objectives of the study. All key experiments supporting the main conclusions of the study were repeated several times in samples prepared from at least 8 different animals obtained from different litters.
Data exclusions	No data were excluded from the analysis
Replication	All experiments were independently conducted at least five times. All attempts to replicate the findings were consistently successful in the present study.
Randomization	Randomization is not relevant to the present study, since experimental animals were obtained from genetically homogeneous colonies and then assigned to different experimental groups according to the treatment/genetic status.
Blinding	The experiments conducted in acute brain slices were performed by the investigator who was blinded to the treatment/genetic status of the study animals. The investigator undertaking behavioral studies was not blinded , but the data analysis was performed by the individuals blinded to the condition, treatment and/or genetic status of experimental subjects and to the identity of the experimental groups. In the metabolomics study, sample separation, mass spectrometry and data analysis were performed by the investigators who were blinded to the

identity of the samples.

Reporting for specific materials, systems and methods

We require information from authors about some types of materials, experimental systems and methods used in many studies. Here, indicate whether each material, system or method listed is relevant to your study. If you are not sure if a list item applies to your research, read the appropriate section before selecting a response.

Materials & experimental systems

n/a	Involved in the study
☐	☒ Antibodies
☒	☐ Eukaryotic cell lines
☒	☐ Palaeontology and archaeology
☐	☒ Animals and other organisms
☒	☐ Clinical data
☒	☐ Dual use research of concern

Methods

n/a	Involved in the study
☒	☐ ChIP-seq
☒	☐ Flow cytometry
☒	☐ MRI-based neuroimaging

Antibodies

Antibodies used

Anti-adenosine A2B receptor antibody (Merck Millipore, Cat # AB1589P); anti-actin antibody (Cell Signaling Technologies, Cat # 3700, clone 8H10D10); anti-chicken antibody AlexaFluor 488 (ThermoFisher, Cat #A-11039); anti-chicken antibody AlexaFluor 568 (ThermoFisher, Cat. # A-11041); anti-rabbit antibody AlexaFluor 568 (Abcam, Cat #175470); rabbit anti-GFAP antibody (Proteintech,Cat # 23935-1-AP); anti-GFP antibody (Aves Labs Cat. # GFP-1020); chicken anti-GFP antibody (Abcam Cat#AB13970); rabbit anti-Iba1 antibody (GeneTex, Cat #GTX100042); anti-rabbit -HRP antibody (Santa Cruz, Cat. # sc-2054); anti-mouse-HRP antibody (Santa Cruz, Cat. # sc-2005); rabbit anti-MBP antibody (ThermoFisher, Cat #MA5-35074, clone 1Z9R5); rabbit anti-NeuN antibody (Abcam, Cat #AB236870, clone EPR21906).

Validation

Anti-adenosine A2B receptor antibody was used for the Western blots which showed reduced expression of A2B receptor in cells of Adora2Bf/f animals transduced to express Cre recombinase. This was expected and consistent with the data showing reduced expression of A2B receptor at mRNA level. Details of antibody validation are available on the manufacturer website: https://www.merckmillipore.com/GB/en/product/Anti-Adenosine-A2b-Receptor-Antibody,MM_NF-AB1589P?ReferrerURL=https%3A%2F%2Fwww.google.com%2F
Anti-GFP antibody was used for immunofluorescence detection and amplification of GFP expression in hippocampal astrcoytes of Adora2Bf/f mice transduced to express Cre recombinase. Details of antibody validation can be found on the manufacture's website: https://www.aveslabs.com/products/anti-green-fluorescent-protein-antibody-gfp; https://www.abcam.com/en-no/products/primary-antibodies/gfp-antibody-ab13970.
Anti-actin antibody was thoroughly validated for the use in western blot in cell/tissue samples of mice and other species. Details of antibody validation can be found on the manufacture's website: https://www.cellsignal.com/products/primary-antibodies/b-actin-8h10d10-mouse-mab/3700). The use of this antibody has been reported in more than 4,000 publications, from which 314 studies used the antibody to perform western blot analysis in Mus musculus samples.
The following primary and secondary antibodies were validated by the manufacturers: rabbit anti-GFAP (https://www.ptglab.com/products/GFAP-Antibody-23935-1-AP.htm); rabbit anti-MBP (https://www.thermofisher.com/antibody/product/MBP-Antibody-clone-ARC0535-Recombinant-Monoclonal/MA5-35074); rabbit anti-NeuN (https://www.abcam.com/products/primary-antibodies/neun-antibody-epr21906-neuronal-marker-ab236870.html); rabbit anti-Iba1 (https://www.genetex.com/Product/Detail/Iba1-antibody/GTX100042); anti-chicken AlexaFluor 488 (https://www.thermofisher.com/antibody/product/Chicken-anti-Rabbit-IgG-H-L-Cross-Adsorbed-Secondary-Antibody-Polyclonal/A-21441); anti-rabbit AlexaFluor 568 (https://www.abcam.com/en-dk/products/secondary-antibodies/donkey-rabbit-igg-h-l-alexa-fluor-568-ab175470).

Animals and other research organisms

Policy information about studies involving animals; ARRIVE guidelines recommended for reporting animal research, and Sex and Gender in Research

Laboratory animals

Young adult C57Bl/6J, Adora2bflox/flox and Aldh1l1Cre/ERT2 mice and their crosses (3-4 mo old) and pups (p2-10) of both sexes; young Sprague-Daweley rats (P21-25) and pups (p2-10) of both sexes.

Wild animals

The study did not involve any wild animals

Reporting on sex

Animals of both sexes were used in the experiments. Data analysis showed no differences in the effects of experimental condition, treatment or genetic status; therefore, the data obtained in male and female animals were pulled. Detailed sex-based analysis was not performed

Field-collected samples

The study did not involve any field collected samples

Ethics oversight

All animal experimentations were performed in accordance with the European Commission Directive 2010/63/EU (European Convention for the Protection of Vertebrate Animals used for Experimental and Other Scientific Purposes) and the UK Home Office

March 2021

Animals (Scientific Procedures) Act (1986) with project approval from the Institutional Animal Care and Use Committee of the University College London.

Note that full information on the approval of the study protocol must also be provided in the manuscript.
